# Performance Evaluation of Malaria Pf/Pv Combo Test Kit at Highly Malaria-Endemic Area, Southern Ethiopia: A Cross-Sectional Study

**DOI:** 10.1155/2020/1807608

**Published:** 2020-09-07

**Authors:** Temesgen Eticha, Tewodros Tamire, Temesgen Bati

**Affiliations:** ^1^Department of Medical Laboratory Sciences, College of Health Sciences and Medicine, Wolaita Sodo University, Sodo, Ethiopia; ^2^Microbiology unit, Tikur Anbessa Specialized Hospital, Addis Ababa, Ethiopia; ^3^School of public health, College of Health Sciences and Medicine, Wolaita Sodo University, Sodo, Ethiopia

## Abstract

**Background:**

Malaria rapid diagnostic tests (RDTs) are alternative diagnostic methods that have enabled reliable biological diagnostic testing in all situations where previously only clinical diagnosis was available. Varying diagnostic accuracy of malaria RDTs makes policymakers confused while choosing malaria test kits for their country.

**Objective:**

The aim of this study was to evaluate the diagnostic performance of currently being used malaria RDT in Southern Ethiopia.

**Methods:**

A cross-sectional study design was conducted from October 1 to December 15, 2016. A total of 160 patients were included in the study. Finger-prick blood sample was obtained from study subjects for the RDT test and microscopic examination. Collected data were entered and analyzed using SPSS version 20.0.

**Result:**

The test kit evaluated had an overall sensitivity, specificity, PPV, and NPV of 97.44%, 93.67%, 93.83%, and 97.37%, respectively, to detect the presence or absence of malaria. Sensitivity and specificity of the kit for *P. falciparum* detection were 63.27% and 94.3% and for *P. vivax* detection were 86.96% and 95.62%, respectively. The agreement between microscopy and RDT for specific identification of malaria species was moderate with a kappa value of 0.568.

**Conclusion:**

The overall performance of the kit was below the WHO standard. Further study on a large sample size is recommended to be carried out in the study area to use the test kit instead of microscopy for malaria diagnosis. Providing training on quality malaria laboratory diagnosis and availing necessary supplies for malaria diagnosis shall also be considered.

## 1. Introduction

In Ethiopia, malaria is the leading cause of morbidity and mortality. Almost 75% of the country is malarious, and an estimated 51 million people (68% of the population) live in areas at risk of malaria [1]. It is a parasitic disease that is transmitted to humans by mosquitoes of the genus *Anopheles* [2]. It is one of the few diseases which have a quick, simple, and accurate biological diagnostic method even in a low-technology setting [3].

The nonspecific nature of signs and symptoms of malaria made a clinical diagnosis of the disease unreliable [4]. Microscopy remains the standard and preferred method of diagnosis compared to other methods despite its apparent drawbacks especially in sub-Saharan African hospitals as it needs significant technical skills and good-quality reagents [4, 5].

RDTs are alternate diagnostic methods which enabled reliable biological diagnostic testing in situations where previously only clinical diagnoses were available, although it has varying diagnostic accuracy across different geographical regions [6, 7]. Their adoption needs to be guided by local sensitivity tests, to detect functional problems due to exposure to heat, humidity, and manufacturing faults [8, 9].

The ability of Care Start™ Malaria Pf/Pv Combo test to diagnose *Plasmodium* malaria was very good, with 99.8% sensitivity and 97.7% specificity, according to the study conducted on acute febrile patients visiting the Felegeselam Health Center, North Ethiopia [10]. Similarly, sensitivity and specificity of the Care Start™ Malaria Pf/Pv Combo test were 98.5% and 98.0% for the diagnosis of *P. falciparum* and *P. vivax* infections according to a study performed in Afar region, Northeast Ethiopia [11].

Even though Arba Minch and adjacent hot rural areas are one of the most highly malarious places in Ethiopia and using malaria RDTs as an alternative diagnostic tool, its performance was not evaluated so far. This study aimed at evaluating the diagnostic performance of malaria RDT by determining its sensitivity and specificity compared with microscopy to help local malaria eradication program presenting the actual performance of currently used malaria RDT.

## 2. Materials and Methods

### 2.1. Study Area

The study was conducted in Arba Minch General Hospital (AGH) and Arba Minch Health Center. Arba Minch town is located in Gamo Gofa Zone of the Southern Nations, Nationalities, and People's Region (SNNPR) about 500 kilometers south of Addis Ababa. It is found at an altitude of 1200–1300 meters above sea level with an average annual temperature of 29.7°C and rainfall of 700 mm [12].

### 2.2. Study Design and Period

A cross-sectional study design was employed from October 1 to December 15, 2016.

### 2.3. Study Participants

The study participants were all individuals who came to AGH and Arba Minch Health Center being suspected of malaria and have a request paper for malaria diagnosis at the respective laboratory unit.

### 2.4. Inclusion Criteria

All age group patients with clinical signs and symptoms of malaria who presented to AGH and health center laboratory department for confirmation during the study periods were eligible for enrollment into the study.

### 2.5. Exclusion Criteria

Clients who do not fulfill the inclusion criteria and refused participation were excluded from the study.

### 2.6. Sample Size and Sampling Technique

Assuming a confidence level of 95%, an error risk of 1.96, an expected minimum sensitivity and specificity of all RDTs of 95%, compared with microscopy according to the World Health Organization [13], and a margin of error of 5% plus an additional 10% of the sample to account for invalid and unclear results, a minimum sample of 80 participants were recruited. According to this assumption, a total of 160 study subjects (80 malaria-positive and 80 malaria-negative) were included in this study.

A convenient sampling technique was employed to obtain those study subjects.

*n*=((*z*^2^)*p*(1 − *p*)/*d*^2^)=80 (including 10% to account for invalid and unclear results), where *n* is the sample size, *z* is the 95% confidence interval (1.96), *d* is the margin of error (5%), and *p* is the sensitivity or specificity (95%).

### 2.7. Data Gathering Procedures

Questionnaires were developed for the purpose of simple sociodemographic data. Formats were used for reporting parasite density and the laboratory result of malaria status by microscopy and RDT. They were prepared originally in English after reviewing relevant literature and then translated to Amharic. Pretest of the questionnaire for the clarity and consistency of questions was performed, and the necessary correction was made based on the findings of the pretest.

### 2.8. Sample Collection and Processing

A blood sample was obtained from eligible study subjects visiting Arba Minch General Hospital and Arba Minch Health Center in the specified study period, having signs and symptoms of malaria, and sent to laboratory diagnosis. From each study participant, a finger-prick blood sample was collected for malaria testing with both Care Start™ RDT (Cat No.G0161 T) and microscope. Two blood films were prepared; the first blood film was processed at a hospital or health center laboratory setup as a routine way, and the result at such facility setup was used as primary reading. Such primary result was recorded separately for the research purpose according to the prelabeled identification number. The second blood film was dried and fixed (the thin film) and examined at Arba Minch College of Health Science teaching laboratory by the principal investigator for quality assurance purposes. Any discrepancy between primary reading and secondary reading was cleared by third-person final reading. The Care Start™ RDT test was performed at the site of sample collection by different laboratory personnel so that the one who did the microscopy do not know the result of the RDT.

### 2.9. Laboratory Procedures

#### 2.9.1. Rapid Diagnostic Testing for Malaria

The available RDT kit was used in this study according to the manufacturer's instructions. It is used for the qualitative detection of antigens produced by *P. falciparum* and *P. vivax*. These antigens are the histidine-rich protein -2 (PfHRP-2) and lactate dehydrogenase (LDH).

#### 2.9.2. Microscopy

Blood smears were stained with 10% Giemsa and examined using 100X oil immersion objective lens of a light microscope by two independent laboratory technologists (one at the hospital/health center and the other at the teaching school laboratory (AMCHS)) who are blinded to each other's results.

#### 2.9.3. Parasite Density

Parasite density estimation was performed by measuring the level of infection in red blood cells by examination of a thin blood film which is confirmed to be more accurate [14]. To quantify malaria parasites against RBCs, parasitized RBCs among 500–2,000 RBCs on the thin smear were counted, and the result is expressed as a percentage of parasitemia.

#### 2.9.4. Methods of Data Analysis

Data collected from this study were entered and analyzed using SPSS version 20. The data include participant demographic information, clinical signs and symptoms, parasite type, previous treatment history, and RDT result. Sensitivity, specificity, positive predictive values (PPVs), and negative predictive values (NPVs) of the Care Start™ Malaria RDT were calculated. Kappa value was calculated to determine the agreement between the results of microscopy and the diagnostic test kit.

#### 2.9.5. Data Quality Management

Fresh blood samples were transferred directly to the sample pad by the provided sample applicator. All Care Start™ malaria test kits were labeled with the patient ID number, and the procedure and the result recording period were according to the manufacturer's instruction. To eliminate observer bias, quality control was performed by the principal investigator at Arba Minch College of Health Science teaching laboratory blindly repeating all test results at the facility level.

## 3. Results

In this study, a total of 160 study participants suspected of malaria were examined for malaria parasites by thick/thin blood smear microscopy and Care Start™ combo RDT test kit. Of the total participants, 89 (55.6%) were males and 71 (44.4%) were females with ages ranging from 1 year to 70 years with a mean age of 24 and a median of 21 (Table 1).

In this study, microscopy and Care Start™ malaria RDT gave similar *P. falciparum* test result for 31 study participants, *P. vivax* test result for 20 study participants, and mixed infection with *P. falciparum* and *P. vivax* for only 5 study participants. As presented in Table 2, surprisingly RDT gave mixed infection for 13 study participants which were only *P. falciparum* by microscopy.

**Figure 1:**
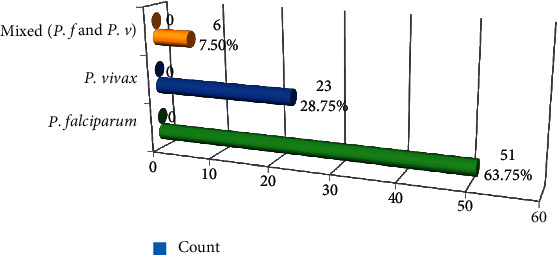
Malaria species prevalence among microscopically confirmed positive cases, Arba Minch, 2016.

Of the entire 160 blood smear slides eligible for analysis, microscopy detected malaria parasites in 80 (50%) blood smear samples. Among microscopically examined blood smear samples of study participants, 51 (63.75%) were infected with *P. falciparum*, 23 (28.75%) were infected with *P. vivax*, and the remaining six (7.50%) had mixed infection with *P. falciparum* and *P. viva*x (Figure 1). No study participants were identified as positive with *P. ovale* and *P. malariae* by malaria microscopy.

Taking a thick blood smear as a gold standard test for malaria, the overall sensitivity and specificity of Care Start™ RDT were found to be 97.44% (95% CI = 91.04%–99.69%) and 93.67% (95% CI = 85.84%–97.91%), respectively. The PPV and the NPV of the device were found to be 93.83% (95% CI = 86.18%–97.97%) and 97.37% (95% CI = 90.82%–99.68%), respectively. The agreement between the light microscopy and Care Start™ RDT to detect the presence or absence of the malaria parasite has a kappa value of 0.877 (Table 3).

The Care Start™ RDT test kit was 63.27% (95% CI = 48.29%–76.58%) sensitive and 94.50% (95% CI = 88.40%–97.95%) specific to detect *P. falciparum* malaria. The PPV and NPV of Care Start™ RDT test kit to diagnose *P. falciparum* were 83.78% and 85.12%, respectively. The corresponding sensitivity and specificity of Care Start™ for the diagnosis *P. vivax* malaria were 86.96% (95% CI = 66.41%–97.22%) and 95.62% (95% CI = 90.71%–98.38%), respectively, with 76.92% PPV and 97.76% NPV. The sensitivity, specificity, PPV, and NPV of Care Start™ for the diagnosis of mixed infection (*P. falciparum* and *P. vivax*) were 83.33%, 91.56%, 27.78%, and 99.30%, respectively. Accordingly, the overall agreement between light microscopy and Care Start™ malaria RDT test kit for specific identification of malaria species has a kappa value of 0.568 (Table 4).

On analysis of the diagnostic performance of the kit used by percent parasitemia, generally, the sensitivity of the kit increased when the percent parasitemia increased in the individual study subject. Assessment of sensitivities at percent parasitemia threshold below 0.049% was not displayed because there is no positive test result by microscopy within that percent parasitemia range. Starting from 1.01% parasitemia up to the maximum percent parasitemia (2.85%), the Care Start™ RDT showed 100% sensitivity. Association of percent parasite load with RDT and microscopy result is presented in Table 5.

Care Start™ Combo test for the detection of *P. falciparum* had a sensitivity of 100% for study participants with percent parasitemia >1.51%. As indicated in Table 6, the sensitivity of the test kit declined from 80.77% (for *P. falciparum*) and 90.91% (for *P. vivax*) to 46.67% and 75.00%, respectively, even though the percent parasitemia increased (Table 6).

## 4. Discussion

Using RDT devices to test the presence or absence of the malaria parasite has many advantages, especially in countries such as Ethiopia where the majority of the population live in rural parts of the country where access to electricity and infrastructures are limited; using RDTs as a diagnostic testing method and treatment monitoring method has a great advantage. Since the performance of these diagnostic testing methods may vary from population to population even in the same country, their diagnostic performance shall be tested locally to check their agreement with the WHO minimal sensitivity and specificity for any RDTs.

In this study, Care Start™ Combo test showed an overall sensitivity, specificity, PPV, and NPV of 97.44%, 93.67%, 93.83%, and 97.37%, respectively. It showed a better performance when compared with the study conducted in Butajira area, south-central Ethiopia, with overall sensitivity and specificity for the diagnosis of malaria of 90.8% and 82.7%, respectively [15]. The variation in the result may be due to the difference in the method used (facility and community survey), with a greater sample size, as they included every febrile case in the study. In contrast, when compared with the study conducted on acute febrile patients visiting Felegeselam Health Center with 99.8% sensitivity and 97.7% specificity [10], our device showed poor overall performance.

Our device has shown almost comparable overall performance with studies carried out in Northwest Ethiopia with an overall sensitivity of 95% and specificity of 94.2% [4], at Serbo Health Center in Jimma Zone, Southwestern Ethiopia, with an overall sensitivity of 95.8% and specificity of 100% [1], and in Northwestern Tigray with a sensitivity of 95.4% [16]. This comparable performance may be due to almost a similar sampling technique, testing procedures, and analysis.

This study has also tried to evaluate the performance of the Care Start™ RDT in detecting different species of malaria parasite. In this study, the sensitivity and specificity of Care Start™ RDT for the diagnosis of *P. falciparum* are 63.27% and 94.3%, respectively, which showed very poor performance when compared to other studies with sensitivity and specificity of 99.4% and 98% in Wondo Genet [17], 98.5% and 98.0% in Afar region [11], and 85.6% and 92% in Oromia Regional State [18], respectively. Variations in test sensitivity between these studies may be due to variations in epidemiologic characteristics of the study population, level of parasitemia, test methodology, and skill of microscopists [19].

Even though the sensitivity and specificity of Care Start™ RDT for the diagnosis of *P. vivax* were better than those for *P. falciparum*, with 86.96% and 95.62%, respectively, it has almost comparable performance with the study carried out at three health centers in Jimma Zone, Oromia Regional State [18]. It showed poor performance when compared with other studies in Wondo Genet (99.4% and 98.2%) [17] and Afar (100% and 99.6%) [11]. Although the manufacturer's instructions were strictly followed, the poor performance of this RDT kit in the current study could be due to the high false-positive results possibly because of the persistent nature of HRP-2 [20] and variation of the geographical regions [7].

Accordingly, even though there was a very good agreement between the light microscopy and Care Start™ RDT to detect the presence or absence of malaria parasite with a kappa value of 0.877, the overall agreement between light microscopy and Care Start™ malaria RDT for specific identification of malaria species is very poor with a Kappa value of 0.568.

The performance of Care Start™ RDT was noted to be significantly influenced by the level of percent parasitemia. In this study, the percent parasitemia ranged from 0.05% to 2.85%. The sensitivity and specificity of the RDT increased when the percent parasitemia increased in the individual study subjects. A similar scenario was observed in other studies [9, 11, 21, 22]. In addition, the chance of malaria RDT kit used to show mixed infection for study participants who had only *P. falciparum* was very high. 13 (8.12%) study participants who had *P. falciparum* only by the standard microscopy were found to have mixed infection by the kit used. A similar finding was observed in a study performed in Madagascar [23] where the kit used (SD Bioline Malaria Ag Pf/Pan) showed mixed infection for those detected as high parasite density *P. falciparum*. This may result in unnecessary drug and resource expenditure in areas where only RDT kit is being utilized as a means of malaria diagnosis.

The main limitation of the study was not using PCR as a reference method, since microscopy has lower sensitivity even when compared with loop-mediated isothermal amplification (LAMP) [24], and obviously, its sensitivity decreases with decreased parasite density.

## 5. Conclusion

Malaria RDT kits may be used in areas where there are no trained personnel, electricity, necessary equipment, and reagents which are necessary for malaria microscopy. Since the performance of RDT may vary with various factors such as loss of integrity of kits during transportation, different climate conditions, and/or inappropriate storage condition, frequent local sensitivity testing shall be carried out.

In this study, the performance of the currently used malaria RDT kit was poor for accurate diagnosis of malaria. It has also a drawback in correctly identifying malaria species of *P. falciparum* and *P. vivax*. So, further studies should be carried out to use the Care Start™ Malaria Pf/Pv Combo test kit instead of microscopy for the diagnosis of malaria. Providing training on quality malaria laboratory diagnosis for the staff and availing necessary supplies for malaria diagnosis shall also be considered. Gamo Gofa Zone Health Department should select and purchase the right type of malaria RDT which displays good performance by conducting area-specific performance evaluation tests.

## Figures and Tables

**Table 1:** Age group distribution of the study participants, Arba Minch, 2016.

Age group	Frequency	Percentage	Microscopy positive result
<5 years	16	10	9
6–15 years	31	19.4	24
16–30 years	72	45	37
31–45 years	25	15.6	9
>45 years	16	10	1
Total	160	100	80

**Table 2:** Comparison of Care Start™ malaria RDT with microscopy for the identification of malaria species, Arba Minch, 2015.

	Malaria species seen by microscopy				
Malaria species detected by RDT		*P. falciparum*	*P. vivax*	Mixed	Total
	*P. falciparum*	31	3	0	34
	*P. vivax*	3	20	1	24
	Mixed	13	0	5	18
	Total	47	23	6	76

**Table 3:** Overall performance characteristics of Care Start™ in comparison with the standard light microscopy, Arba Minch, 2016.

Performance characteristics	Care Start™ malaria
Sensitivity (95% CI)	97.44% (91.04%–99.69%)
Specificity (95% CI)	93.67% (85.84%–97.91%)
Positive predictive value (95% CI)	93.83% (86.18%–97.97%)
Negative predictive value (95% CI)	97.37% (90.82%–99.68%)

**Table 4:** Performance characteristics of Care Start™ by species identified in comparison with the standard light microscopy, Arba Minch, 2016.

Parasite type	Sensitivity (95% CI)	Specificity (95% CI)	PPV (95% CI)	NPV (95% CI)
*P. falciparum*	63.27% (48.29–76.58)	94.50% (88.40–97.95)	83.78% (67.99–93.81)	85.12% (77.51–90.94)
*P. vivax*	86.96% (66.41–97.22)	95.62% (90.71–98.38)	76.92% (56.35–91.03)	97.76% (93.60–99.54)
Mixed infection	83.33% [35.88–99.58%]	91.56% (86.00–95.43)	27.78% (9.69–53.48)	99.30% (96.14–99.98)
Overall performance	97.44% (91.04–99.69)	93.67% (85.84–97.91)	93.83% (86.18–97.97)	97.37% (90.82–99.68)

**Table 5:** Association between the sensitivity of Care Start™ and percent parasitemia, Arba Minch, 2016.

Range of percent parasitemia	Sensitivity (95% CI)
<0.049%	ND
0.05%–0.50%	97.44% (86.52–99.94)
0.51%–1.00%	96.15% (80.36–99.90)
1.01%–1.50%	100% (59.04–100)
1.51%–2.00%	100% (15.81–100)
>2.01%	100% (39.76–100)

**Table 6:** Sensitivity and specificity of Care Start™ Malaria Pf/Pv Combo test for the diagnosis of malaria species infections at different levels of percent parasitemia, Arba Minch, 2016.

Parasite type	Sensitivity		Specificity	
Range of percent parasitemia	*P. falciparum*	*P. vivax*	*P. falciparum*	*P. vivax*
<0.049%	ND	ND	ND	ND
0.05%–0.50%	80.77%	90.91%	92.31%	88.89%
0.51%–1.00%	46.67%	75.00%	81.82%	94.74%
1.01%–1.50%	0%	100%	100%	100%
1.51%–2.00%	100%	ND	ND	ND
>2.01%	100%	100%	100%	100%

## Data Availability

Data used to support the finding of this study are available from the corresponding author upon request.
